# Geometry of plastic deformation in metals as piecewise isometric transformations

**DOI:** 10.1038/s41598-024-70077-3

**Published:** 2024-08-18

**Authors:** Yan Beygelzimer, Alexander Filippov, Dmytro Orlov

**Affiliations:** 1 Donetsk Institute for Physics and Engineering Named After O.O. Galkin, NASU, Kyiv, Ukraine; 2 Department of the Functional Morphology and Biomechanics, University of Kiel, Kiel, Germany; 3 Division of Mechanics, Materials and Component Design, LTH, Lund University, Lund, Sweden

**Keywords:** Plastic deformation, Deformation mechanisms, Piecewise isometric transformation, Severe plastic deformation, Mechanically assisted diffusion, Mechanical properties, Metals and alloys

## Abstract

Deformation mechanisms of crystalline solids has been the subject of research for more than two centuries. The theory of dislocations dominates modern views but still has significant gaps demanding the introduction of additional concepts for the coherent quantitative description of physical phenomena. In this work, we propose a coherent geometric description of motion and deformation in crystalline solids as piecewise isometric transformations (PWIT). The latter only includes operations that, similar to interatomic spacing in crystalline lattice, do not alter distances between reference points, i.e. translations, rotations and mirror reflections. The difference between solid-body translations and plastic deformations is that the isometric transformations have discontinuities that in real-life materials realise through dislocations (termination of shifts), disclinations (termination of rotations), and twins (mirror reflections). The conceptual description of plastic deformations as PWIT can be useful for the better description of physical phenomena, proposing new hypothesis, and for developing predictive analytical models. In this paper, the use of this conceptual description enables proposing new hypothesis about the nature of such interesting phenomena in severe plastic deformation as (i) stationary ‘solid state turbulence’ stage in high pressure torsion, and (ii) rate of mass transfer (mechanically assisted diffusion) in simple-shear deformation.

## Introduction

In the theoretical analysis of plastic deformation, quite a few approaches have been developed by now. These approaches can be grouped based on the subject of investigation, and in the order of increasing scale include: defects of lattice structure^1,2^, fields representing such defects in continual and calibration theories^3^, slip systems in self-correlative models of polycrystals^4,5^, crystal aggregates in crystal plasticity^5^, continua with microstructure in homogenisation methods^6,7^, continua in classical and gradient theory of plasticity^8,9^.

Such a variety of approaches, reflects the objective complexity of plastic deformation process. It forces researchers to focus on key aspects in specific problems while simplifying other elements for ensuring validity in a particular approach. For instance, the modelling of crystalline structure defects allows studying elastic–plastic transition, which is critically important in structural stability problems^10^. Continuum material models allow studying large plastic deformations calculating the parameters of power and net shape evolution in metal forming^9,11^. Integrated approaches are used in the studies of metal microstructure and texture evolution in large plastic deformations^4–7^.

Each of the approaches is bound to specific geometric descriptions. For instance, Riemannian space (or manifold) with non-zero curvature is used in the continuum theories describing the defects of crystalline structure. At the same time, most of the theories of plasticity are based on the use of Euclidean space with zero curvature. In the latter, functions describing the evolution of objects’ geometry are considered smooth, which is bound to the introduction of translation gradients in the consideration. This leads to the use of affine transformations for describing deformations^12^.

In this work, we propose a geometric approach for describing plastic deformations in crystalline materials, which is based on discontinuous isometric transformations. Our approach is based on the assumption that large plastic deformations occur without changing the crystal lattice of a material. This assumption violates the Cauchy-Born rule commonly accepted in the case of small strains^13^. Similar ideas have already been expressed in ref.^14^ where plastic deformation is argued to be the source of dislocations without the deformation of crystal lattice. In that work, the resulting Burgers vector of inhomogeneous macroscopic plastic deformation with a large number of dislocations is introduced, and the mathematical apparatus for its calculation is developed. This is a good basis for the development of large deformations models based on the idea of crystal lattice invariability.

In this work, the implications of the assumption of crystal lattice invariability under large plastic deformations elaborated from the standpoint of Piecewise Isometric Transformations. The proposed approach is illustrated by the plausible explanations of two unexplained phenomena found in High Pressure Torsion (HPT)^15^, one of the main methods of Severe Plastic Deformation (SPD)^16,17^.

## Plastic deformation of metals as piecewise isometric transformations (PWIT)

Metallic material is typically a polycrystal consisting of a giant quantity of atoms that shift in space to realise plastic deformation. Mathematically, this can be expressed as the transformation of their coordinates:

1 $${\varvec{m}}={\varvec{G}}\left({\varvec{M}}\right)$$

where ${\varvec{M}}$ and ${\varvec{m}}$ are the vectors of initial and final coordinates, respectively, and ${\varvec{G}}$ is a mapping operator.

Due to the enormous dimensionality of a problem, it is virtually impossible to find the transformation (1) and to relate it to the external loads applied to a specimen. Therefore, the process of deformation is typically considered at several scale levels.

At the macroscopic level, metals are modelled as a continuum, the deformation of which leads to a change in the lengths of material segments and the angles between them. This is described by the affine transformation of coordinates:

2 $$d{\varvec{x}}={\varvec{F}}\left({\varvec{X}}\right)d{\varvec{X}}$$

where ${\varvec{X}}$ and ${\varvec{x}}$ are the coordinates of a material point in the body before and after deformation, respectively; ${\varvec{F}}\left({\varvec{X}}\right)=d{\varvec{x}}/d{\varvec{X}}$ is a deformation gradient.

Transformation (2) represents the mapping (1) at the macroscopic scale and does not take into account the micro-scale effects. Accounting for the crystalline structure of metals is based on decomposition ${\varvec{F}}={{\varvec{F}}}^{*}\bullet {{\varvec{F}}}^{p}$, where ${{\varvec{F}}}^{*}$ is a term describing the deformation gradient caused by the elastic deformations and the rotations of the crystal lattice, and ${{\varvec{F}}}^{p}$ is a term describing the lattice preserving plastic flow^18^.

Typically, the elastic distortions of the crystal lattice are small during shear in metals leading to the elastic deformation of the crystal lattice at the order of ${10}^{-3}$. Exceptional cases having a small number of lattice defects, or their insufficient mobility, are very small samples, e.g. nano- crystals and whiskers, and specialty superelastic and amorphous alloys. Assuming our case typical, the mapping (1) can be considered isometric, as it does not change the lengths of segments and angles^12^. Such transformations include displacement, rotation, and symmetric reflection.

According to the isometric transformation theorem^19^, a continuous mapping isometric in a small neighbourhood is also isometric in the entire domain. Therefore, in order to lead to a perceptible change in lengths at a macroscopic scale, the mapping (1) must have discontinuities^20^ and to belong to the class of piecewise isometric transformation (PWIT)^21^. Such transformations can be found in the analysis of various systems and phenomena. For instance, they can describe the process of folds formation on the surface of shells^22^, mixing of granular materials^23^, and the specifics of dynamic systems^21,24^.

In the case of small elastic distortions of the crystal lattice, it is possible to neglect in Eq. (2) the elastic part and to take ${\varvec{F}}={{\varvec{F}}}^{p}$ . Then, it follows that the mapping ${{\varvec{F}}}^{p}$ belongs to PWIT.

**Figure 1:**
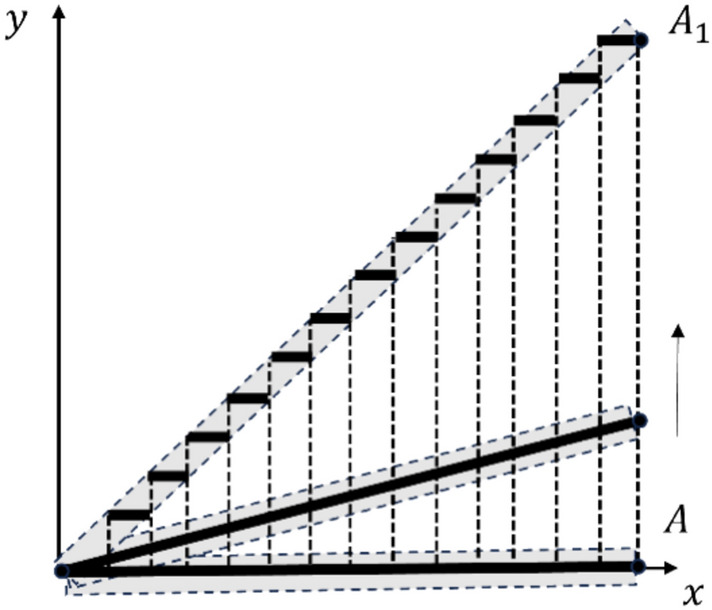
Schematics illustrating the “elongation” of a fibre through PWIT.

Figure 1 shows a simple model illustrating the possibility of changing a fibre length through PWIT.

Shifting point A in the vertical direction leads to an increase in the length of fibre OA, i.e. to a deviation from isometry. When it reaches a threshold value (point A reaches position A_1_), several discontinuities form at points ${x}_{i}$ $\left(i=1,...,4\right)$ that restore the isometry at a micro-scale while allowing the entire fibre to “elongate”. Further upward movement of point A will lead to the sequential repetition of specified steps: deviation from the isometry followed by its local restoration through the emergence of new discontinuities in the isometric transformation.

From the above reasoning, it follows that the mapping ${\varvec{G}}$ can be represented as the product (sequential action) of step-by-step mappings $\Delta {{\varvec{G}}}_{i}$:

3 $${\varvec{G}}={\prod }_{i}\Delta {{\varvec{G}}}_{i}$$

where $i$ is the number of a deformation step, and.

4 $$\Delta G_{i} = \Delta P_{i} \Delta E_{i}$$

where $\Delta {{\varvec{E}}}_{i}$ are affine transformations corresponding to the infinitesimal elastic deformation of crystal lattice; $\Delta {{\varvec{P}}}_{i}$ are PWITs that return the crystal lattice parameters to near-original values and allow the representative volume of a material to realise large deformations.

This allows us to assume that plastic deformation in metals realises through the periodic localised accumulation of elastic energy in a crystal lattice followed by its relaxation through the creation of isometric discontinuities.

To connect this vision with the commonly accepted mechanisms of plastic deformation in metals, let us consider the following question. What are the physical mechanisms realising the PWIT? The answer is following. At the atomic scale, the discontinuities associated with displacements, rotations, and symmetric mirror reflections are realised through dislocations, disclinations, and twins, respectively. At the meso- and macro-scopic scales, the mechanisms realising PWITs include shifts along unstable high-angle boundaries, shear bands, Lüders bands, and fractures.

For the modelling of deformation using PWIT-based method, a ‘Peridynamic’ method^25^ can be used. Traditional methods of solid mechanics are not appropriate for such modelling since they require the description of displacements by smooth functions due to the need of calculating spatial derivatives. By contrast, the Peridynamic approach makes it possible to solve problems with discontinuities, since it does not use spatial differentiation. The Peridynamic modelling has already been used by one of us for modelling fracture in Harmonic-Structure materials^26^. Nevertheless, in this work we use the similar method of Brownian dynamics, which also allows describing displacement fields with discontinuities to illustrate the qualitative features of plastic deformation based on PWIT.

Another feasible approach for modelling crystal lattice evolution is molecular dynamics^27^. However, standard molecular dynamic simulation operating with the particles of atomic size is unfortunately limited either by extremely small times and size of the systems or by very time-consuming program runs. Using effective “particles” (movable automata) with simply regulated and physically understandable interactions as well as other parameters of the model makes it more transparent and allows direct, almost real-time, observations of the process in the course of numerical experiments with easily varied conditions.

## Heuristic model of plastic deformation

In the analysis of complex systems, so-called ‘heuristic’ models are proven to provide excellent insights^28–30^. They grasp only the main features of a phenomenon and enable studying it in various circumstances facilitating a better understanding.

Recently, we developed a heuristic computer model representing metal as the dense system of interacting particles, which may be perceived rather naïve from the perspectives of physics and mechanics, but it enables studying plastic deformation within the framework of our geometric approach. It describes the partitioning of a particle system into ordered segments and reveals that the shear in such a system takes place by the movement of isometric discontinuities within it. This model has already been used for the analysis of solid lubricants efficiency^31,32^, and for studying ‘solid-state turbulence’ in the process of high-pressure torsion^33^. Full description of the model can be found in ref.^34^, while brief introduction of the model is provided below.

The model is based on Brownian dynamics and describes a system of *N* classical particles characterized by a coordinate ${{\varvec{r}}}_{i}$ and a conjugate pulse ${{\varvec{p}}}_{i}$. Each particle interacts with all the others by a short-range potential $U(|{{\varvec{r}}}_{i}-{{\varvec{r}}}_{j}|)$ according to the following Hamiltonian:

5 $$H({{\varvec{r}}}_{i},{{\varvec{p}}}_{i})={\sum }_{i=1}^{N}\frac{{{\varvec{p}}}_{i}^{2}}{2{m}_{i}}+\frac{1}{2}{\sum }_{i,j=1}^{N}U(|{{\varvec{r}}}_{i}-{{\varvec{r}}}_{j}|).$$

For the sake of certainty and stability of the computational procedure, it is convenient to model the interaction between particles with a pair of Gaussian potentials^34^:

6 $$U(|{{\varvec{r}}}_{i}-{{\varvec{r}}}_{j}|)={C}_{ij}\mathit{exp}\left[-{\left(\frac{{{\varvec{r}}}_{i}-{{\varvec{r}}}_{j}}{{c}_{ij}}\right)}^{2}\right]-{D}_{ij}\mathit{exp}\left[-{\left(\frac{{{\varvec{r}}}_{i}-{{\varvec{r}}}_{j}}{{d}_{ij}}\right)}^{2}\right]$$

where ${C}_{ij}$ and ${D}_{ij}$ are the intensities of attraction and repulsion, and ${c}_{ij}$ and ${d}_{ij}$ the widths of a repulsive core and the radius of attraction, respectively. For the existence of an equilibrium minimum, the quantities of ${C}_{ij}$ and ${D}_{ij}$ are usually such that ${C}_{ij}>>{D}_{ij}$, and the radii of interaction satisfy the opposite inequality, although not necessarily a strong one: ${c}_{ij}<{d}_{ij}$.

The number of the automata (particles) in the model is fixed. Each of them has prescribed mass and all other properties that conserve independently on their redistribution during diffusion. Periodic boundary conditions used in the simulation support the conservation. Technically, it is organized as follows. Every particle leaving the system returns to it from opposite boundary with the same velocity. Besides, it interacts with all the particles from its direct vicinity as well as with the particles from the opposite side of the system. These interactions are counted exactly in the same manner as direct interacting with the particles inside the system (so called “toroidal boundary conditions”). In some sense, one deals here with a periodically continued quasi-infinite system. This procedure ensures mass and momentum conservation and was checked in a number of our previous works devoted to the close problems, e.g.^33^.

Initially, the particles are randomly placed within a rectangular area with lengths $[0,{L}_{x}]$ and $[0,{L}_{y}]$ along each direction. To analyse the problem of deformation by shear, which is key in our study, it is reasonable to apply periodic boundary conditions along horizontal axis. Thus, a particle going beyond the interval $[0,{L}_{x}]$ returns from the opposite end, and vectors ${{\varvec{r}}}_{i}-{{\varvec{r}}}_{j}$ connect both particles within the interval $[0,{L}_{x}]$ and their “images” at the opposite end of a “coiled” system.

Along vertical axis, the system is bounded by two plates with coordinates $y=0$ and $y={L}_{y}$ with reflective boundary conditions. The latter can be realised by steep exponential walls.

${U}_{up}=C\mathit{exp}[(y-{L}_{y})/c]$ and ${U}_{down}=C\mathit{exp}[-y/c]$ and the choice of force signs ensuring repulsion from them. The greater the inequality $C>>{C}_{ij}$ and the smaller the distance of potential decay $c<<{c}_{ij}$, the stiffer and steeper the walls with regard to characteristic forces and distances between the particles.

It should be noted that the apparent simplicity of the equations of motion

7 $${m}_{i}\frac{\partial {{\varvec{v}}}_{i}}{\partial t}=-\frac{\partial H({{\varvec{x}}}_{i},{{\varvec{p}}}_{i})}{\partial {{\varvec{p}}}_{i}}={{\varvec{f}}}_{i}$$

is somewhat deceptive since in fact it implies the summation at each step for all possible neighbours, including those very distant, since their mutual positions change. This should be carried out in each direction including the particle “images” obtained through the periodic boundary conditions. Furthermore, masses ${m}_{i}$, may be different for different particle kinds in other cases when the system is composed of two (or more) subsystems, e.g. in the case of multi-phase materials. Finally, in the cases when one or more boundaries (or the entire volume) come into contact with a ‘thermostat’, the boundary conditions should be replaced with more realistic ones. Namely, if the temperature of the thermostat at a given boundary (or within the system) *T* is non-zero, in the simplest case one should add a random (generally speaking, independently distributed along both *x* and *y* coordinates).

*δ*-correlated Langevin source $\xi (t,x,y)$, which according to the fluctuation-dissipative theorem is related to temperature *T* by the following relations:

8 $$\left\langle {\xi \left( {t,x_{i} ,y_{i} } \right)} \right\rangle = 0;\left\langle {\xi \left( {t,x_{i} ,y_{j} } \right)\xi \left( {t\prime ,x_{j} ,y_{j} } \right)} \right\rangle = D\delta \left( {t - t\prime } \right)\delta_{{i_{j} }} ,$$

where $D=2\gamma {k}_{B}Tm/\Delta t$, ${k}_{B}$ is the Boltzmann constant, $\gamma$ is the dissipative constant taking into account the exchange of energy with the thermostat, *m* is a mass, and $\Delta t$ is the discrete step of computational procedure in time. Also considering that particles exchange momentum upon interaction, it is necessary to take into account yet one more channel of energy dissipation, which works in the direction of equalising the relative velocity of particles at a distance ${{\varvec{c}}}_{{\varvec{v}}}$ close to the equilibrium ${f}_{i}^{{\varvec{v}}}\sim {\sum }_{j=1}^{N}({{\varvec{v}}}_{i}-{{\varvec{v}}}_{j})\mathit{exp}\left[-[({{\varvec{r}}}_{i}-{{\varvec{r}}}_{j})/{c}_{v}{]}^{2}\right]$ with another dissipative constant *η*. So, as a result, we have the equations of motion in the form:

9 $$m_{i} (\partial v_{i} )/\partial t = f_{i}^{r} - \eta f_{i}^{v} - \gamma v_{i} + \xi (t,r_{i} )$$

To solve these dynamic equations with the boundary conditions described above, we integrate them at each step in time using a difference scheme, which conserves the energy of the system (if such is not supplied from the outside by temperature or mechanical sources)^27^. In what follows, we are only considering problems where the temperature of the thermostat *T* is negligible compared to the characteristic energies of the system’s potentials.

It can be verified^35^ that in the limit of high density of kinetic energy ${E}_{kin}/{C}_{ij}>>1$, the system described by Eqs. (9) behaves like a gas of almost free-flying particles, slightly perturbed by weak interactions. At the opposite limit of low energy density, ${E}_{kin}/{C}_{ij}<<1$, the system spontaneously forms a crystal lattice with a constant *a* determined by the equilibrium distance given by the balance of interactions (6).

In such a state, each particle oscillates within the minima of the potential well formed by its neighbours. Thus, its motion can be described by the harmonic Hamiltonian, which is slightly perturbed by the nonlinear terms. In this sense, the system deforms elastically. In the gas state, the system is on average isotropic whereas in the solid state, with an isotropic potential $U(|{{\varvec{r}}}_{i}-{{\varvec{r}}}_{j}|)$, it has a hexagonal shear symmetry with the constant *a*. Within both these limits, the system exhibits a regular dynamic behaviour. However, due to the difference in symmetry, there is a transition through a mixed disordered state^27,36^, which is a viscous liquid, taking into account the dissipations.

In the summary of this section, let us make a note regarding the values of model parameters in the numerical experiments discussed below. Their main goal was to identify qualitatively different options in the system behaviour, and not to study quantitatively the deformation of specific materials. Therefore, the parameters varied in a wide range up to ten times difference between “soft” to “hard” materials. Approximate values of parameters and their relationship with the physical characteristics of materials can be found elsewhere^37^.

## Analysis of PWIT in a simple shear within the heuristic model framework

**Figure 2:**
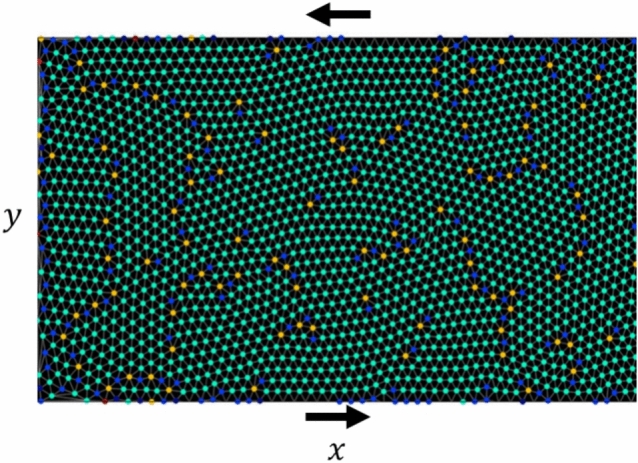
Dense system of strongly interacting particles in the initial state. Separate disoriented domains with hexagonal structure are clearly visible. Green, yellow, and blue colours indicate particles that have 6, 7, and 5 neighbours, respectively. Particles of the latter two types are associated with an elevated energy state (displaced into non-hexagonal positions) and are located along the boundaries of the domains. The arrows at the top and bottom show the directions of subsequent movement of the plates.

Let us study the simple shear of a dense system of strongly interacting particles (${E}_{kin}/{C}_{ij}<<1$) between two parallel plates. First, a number of particles are randomly scattered over a rectangular area with lengths $[0,{L}_{x}]$ and $[0,{L}_{y}]$ at a temperature close to zero, where they are ‘relaxed’, i.e. allowed to settle in order to minimise total energy in the system. As a result, they form a structure consisting of multiple domains, “crystallites”, with differently oriented lattices, see Fig. 2. In this figure, the particle colours represent the number of their nearest neighbours: those with six neighbours are green, with more or less are shifted to red and blue spectrum regions, respectively. The overwhelming majority of particles have six neighbours in-plane. Such an arrangement maximizes the packing density in a plane and minimizes the potential energy of a system, according to the potential in Eq. (6). The hexagonal lattice is unique in its combination of high symmetry and stability and works as an ideal arrangement of particles in a plane, e.g. {111} in FCC lattice or (0001) in HCP. But the system never reaches such a state in reality, as this is prevented by excessively long search time for the absolute minimum as well as random fluctuations of particles due to temperature and kinetic energy from other sources (shear in particular). Therefore, particles with the “wrong” environment appear, which is shown in red and blue colours in Fig. 2.

The system in Fig. 2 is being sheared from above and below in opposite directions.

**Figure 3:**
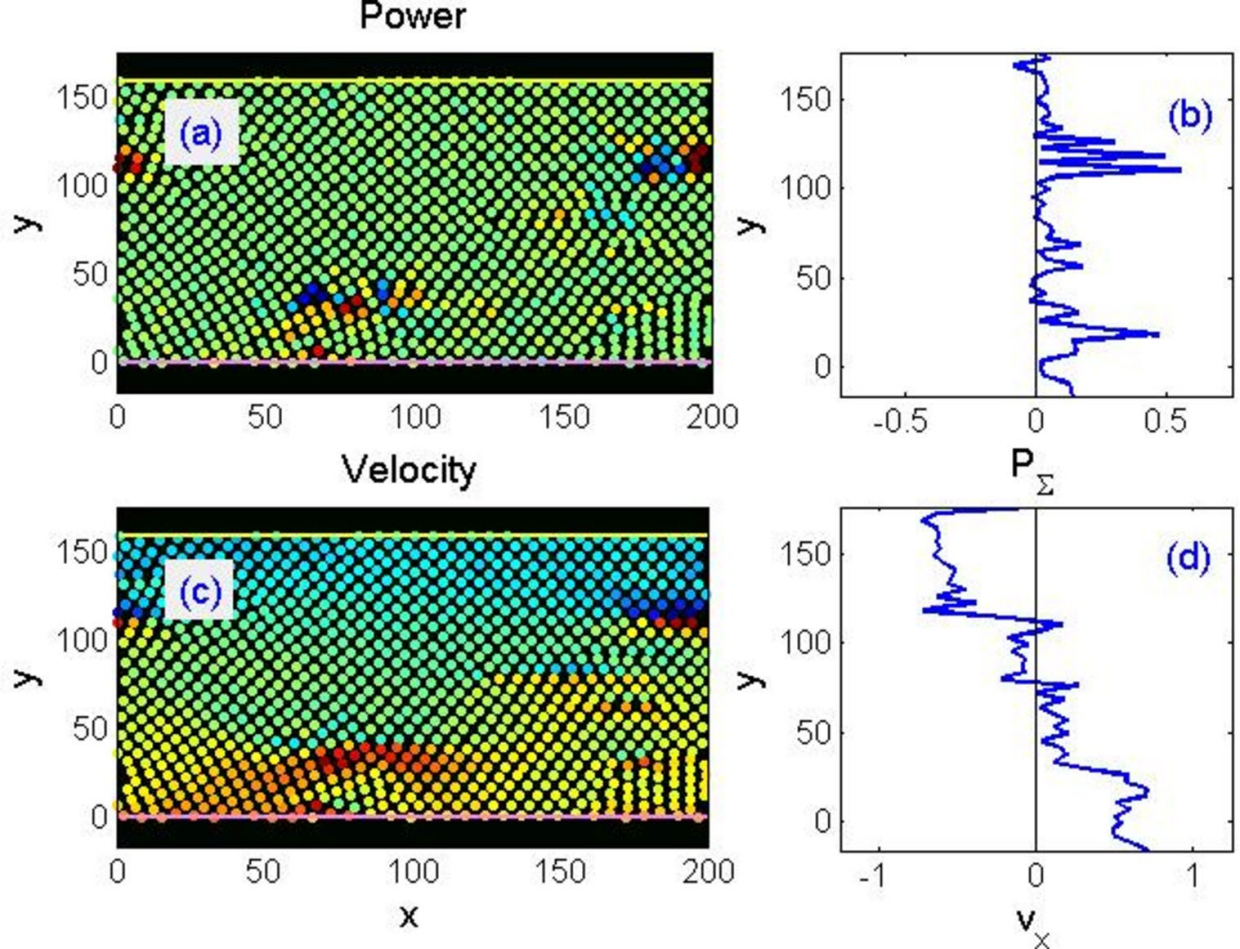
Typical instantaneous distribution of power consumption (**a**, **b**) and velocities (**c**, **d**) under shear strain of a rigid single-component system, shown in the conditional colours of circles representing individual particles. In (**a**, **c**), higher values correspond to red tones, while lower to blue. The same quantities integrated along the horizontal coordinate are shown in panels (**b**) and (**d**), respectively. Correlation between the features of the two- and one-dimensional distributions of both quantities, described in the main text, is clearly visible.

It is convenient to visualise the instantaneous velocities of particles (for example, their horizontal components) with conditional colours. This leads to the formation of a kind of velocity “density map” of a type shown in Fig. 3. It is important to note that besides information about the continual density of a particular quantity (obtained in the limit of a large particles number), it retains information about the microstructure. In particular, about the presence or absence of symmetry, or the different orientation of ordered domains typically visible in figures but requiring quantitative confirmation.

Under external loads, the particles start moving tending to follow the upper and the lower plates. If the flow is laminar, the velocity distribution along axis $y$ should become a smooth monotonic curve over time (in fact, almost a straight line). However, if a “solid body” created in the way described above is divided into disordered domains, i.e. polycrystals with random grain orientations, this is generally impossible. At first, it is deformed elastically, the particles smoothly follow the displacement of plates, and the curve of velocity decay in depth ${v}_{x}(y)$ is smooth. However, bonds between some of the nearest neighbours begin breaking upon exceeding a critical shift, and other neighbours become nearest at some point. Although nominally all particles in the model interact with all others, in practice they tend to follow only immediate surroundings. Namely, a lattice fragment moves as a whole locally and jumps from one crystal plane to another only at the points of contact between differently ordered domains. Such a behaviour of the model corresponds to the above-mentioned property of metals to execute large deformations along grain boundaries by PWIT.

A typical movement of this kind is illustrated in Fig. 3. We intentionally selected a small system for the illustration, so that tracing the orientations in disordered domains would be possible both in a static pattern and in dynamics. Red and blue circles on the velocity distribution (c) represent positive and negative projections of horizontal velocity, respectively, while the intermediate tones correspond to the intermediate speeds in-between. On the profile of longitudinal velocity ${v}_{x}(y)$ averaged along horizontal axis, steps are clearly visible, which indicates a distinct layer-wise movement of the system.

Contrasting spots and bands on the velocity map Fig. 3c clearly depict the instantaneous bursts of particle velocities (in both directions) in the vicinity of interfaces between near-constant velocity regions, where atoms switch from one crystalline plane to another. These places should be interpreted as discontinuities (features) of isometry. They are expected to have the highest level of energy dissipation. It is possible to calculate the work produced by opposing forces acting on each of the system particles per unit of time, ${p}_{i}={{\varvec{f}}}_{i}{{\varvec{v}}}_{i}$ , and to construct its map in conventional colours in the same way as for speeds. Comparison of panels (a) and (c) in Fig. 3 reveals a clear correlation between the locations of isometric discontinuities and power consumption. Integration along horizontal axis, panel (b), also gives an apparent correspondence between the discontinuities of laminar flow bands and the power maxima.

As the plates move, this picture continuously evolves, stresses in the system rise and drop, as the layers move and the energy transfers between them by means of waves propagating through the system. For the static presentation of events, one can also record the history in the form of a space–time map of velocity distributions ${v}_{x}(t,y)$. Summing up the density ${p}_{i}={{\varvec{f}}}_{i}{{\varvec{v}}}_{i}$ throughout the system, we obtain the full power $2V{F}_{ext}(t)=P(t)={\sum }_{i=1}^{N}{{\varvec{f}}}_{i}{{\varvec{v}}}_{i}$ dissipated by the pair of external loads to maintain the movement of the plates at a constant velocity *V* = *const*. Which gives the measured value ${F}_{ext}(t)$.

**Figure 4:**
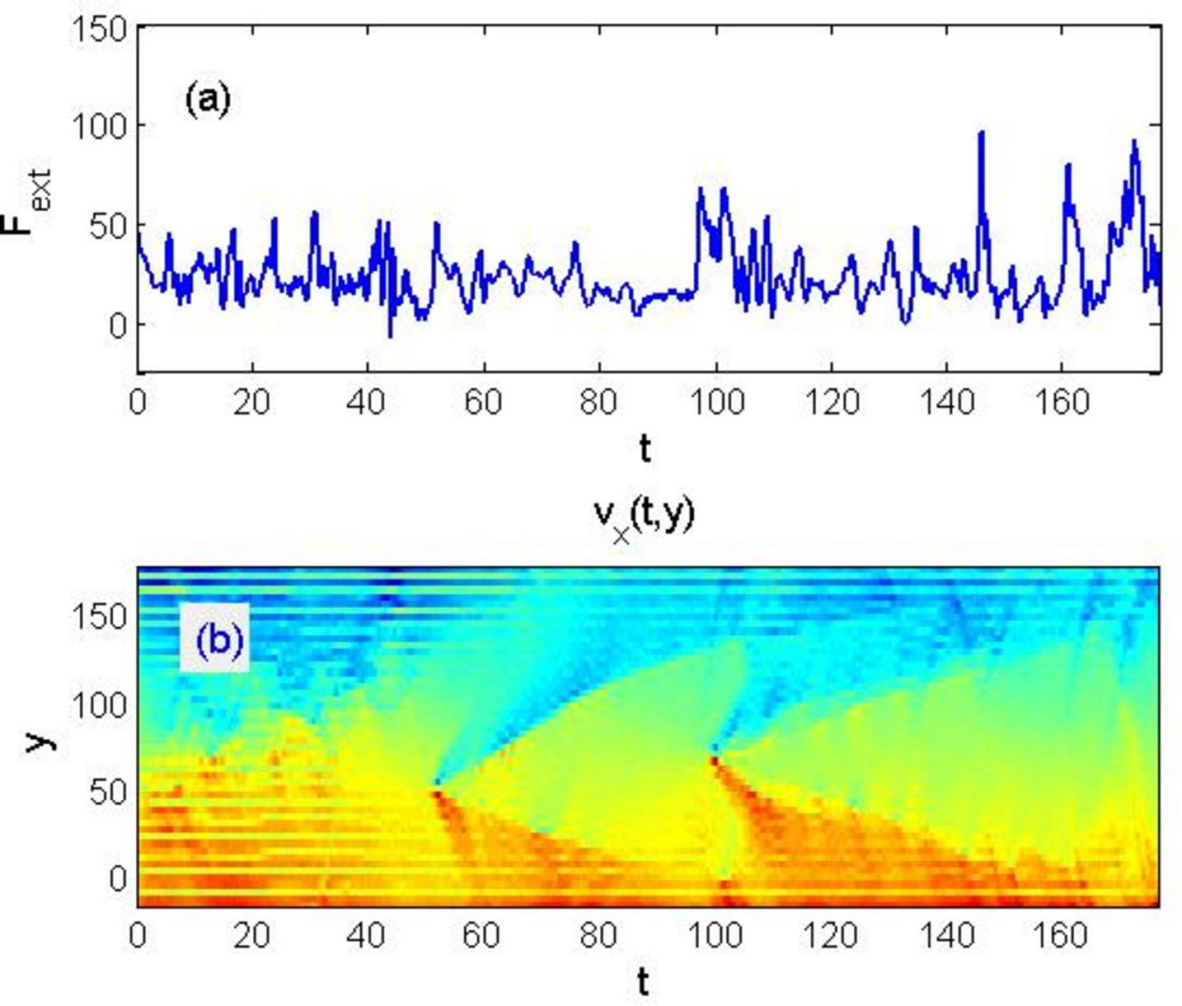
Time-dependencies of (**a**) total force maintaining a constant relative velocity of the plates, and (**b**) accumulated spatio-temporal map showing the history of evolving velocity integrated along the horizontal coordinate v_x (t,y). The propagation of waves associated with the rearrangement of domains accompanying the plastic deformation of the system is clearly visible.

Correlation between this magnitude and the propagation of excitations associated with the rearrangements of layered body motion is obvious in Fig. 4. It should be noted that the dependence of force on time ${F}_{ext}\left(t\right)$, Fig. 4a, resembles the typical dependence of force in the “stick–slip” motion of mutual friction on randomly rough surfaces^38^.

It is useful to compare these results with the behaviour of a much “softer” system. For the sake of comparison, we calculated the same quantities for the case of both the repulsion ${C}_{ij}$ and attraction ${D}_{ij}$ intensities five times lower. In this case, the equilibrium distance between the particles remains the same, but the depth of corresponding energy minimum is much smaller. Since work carried out by the plates during motion brings energy into the system even at zero thermostat temperature in the presence of dissipation, the lattice can be easily “melted” at least in the places of developing local stresses between the layers. In such a system, the long-range order becomes lost quickly, and the atoms easily change neighbours. This behaviour is analogous to a viscous liquid.

**Figure 5:**
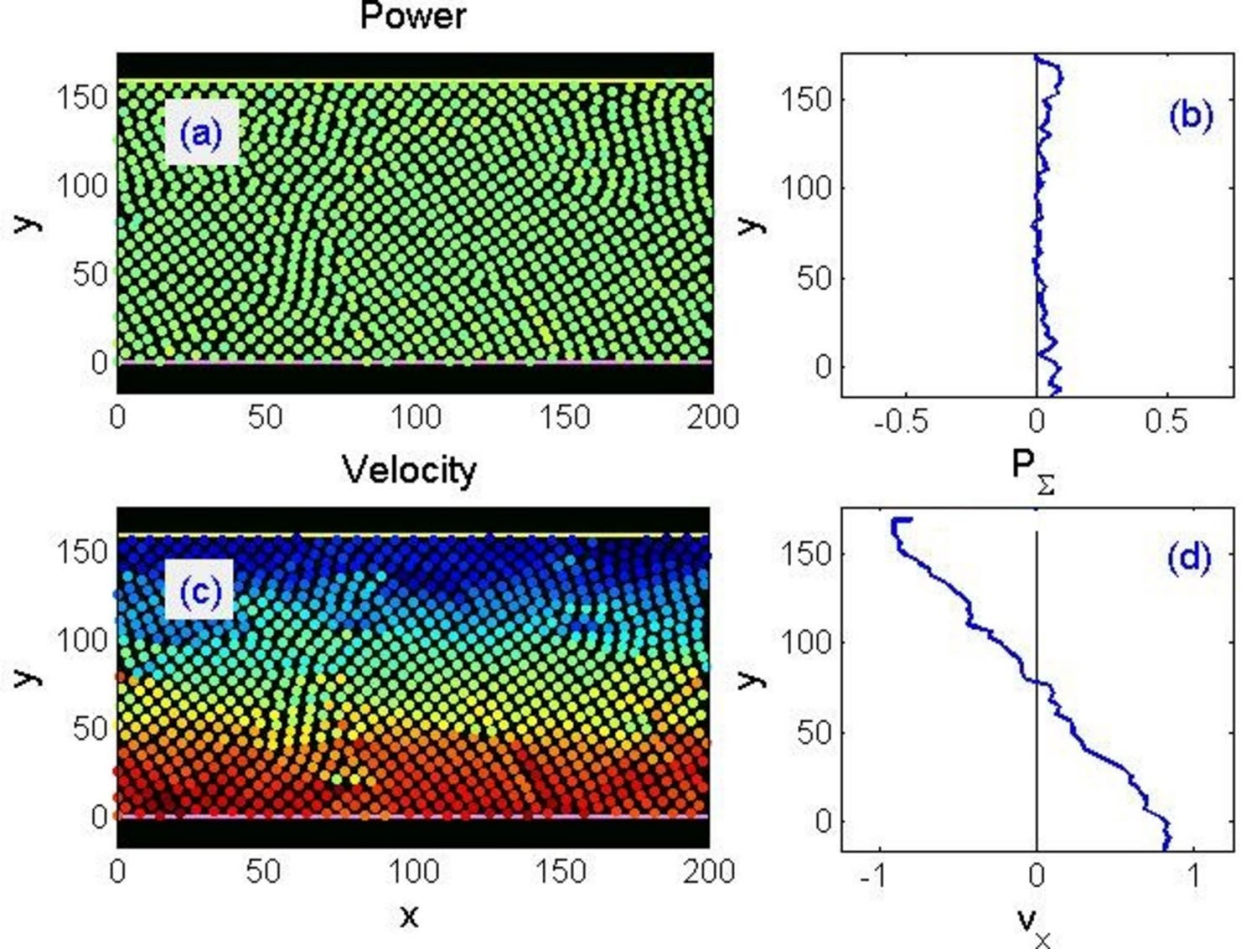
Typical distribution of velocity in a soft system, illustrated in a way similar to Fig. 3. The distribution of velocities along vertical coordinate v_x (y) is monotonic and almost linear, except for small fluctuations, while the power consumption P(y) is relatively low.

The results of a numerical experiment presented in Fig. 5 confirm this by showing a typical velocity distribution in a soft system. Almost laminar flow with the exception of small fluctuations can be seen. The distribution of velocities along vertical coordinate is monotonic and almost linear, except for a slight flattening of the ${v}_{x}(y)$ dependence near the plates. The power consumption $P(y)$ is relatively low and increases just slightly near borders.

Recalling the rigid system, including the analogue of “stick–slip” behaviour revealed by the model, it is reasonable to wonder why this effect is not found, or negligible, in experiments? We believe this is primarily because experiments are typically carried out on macroscopic objects where individual bursts of energy consumption in various sample areas cancel each other out, and the collective behaviour of a system becomes “self-averaged”, or homogenised.

**Figure 6:**
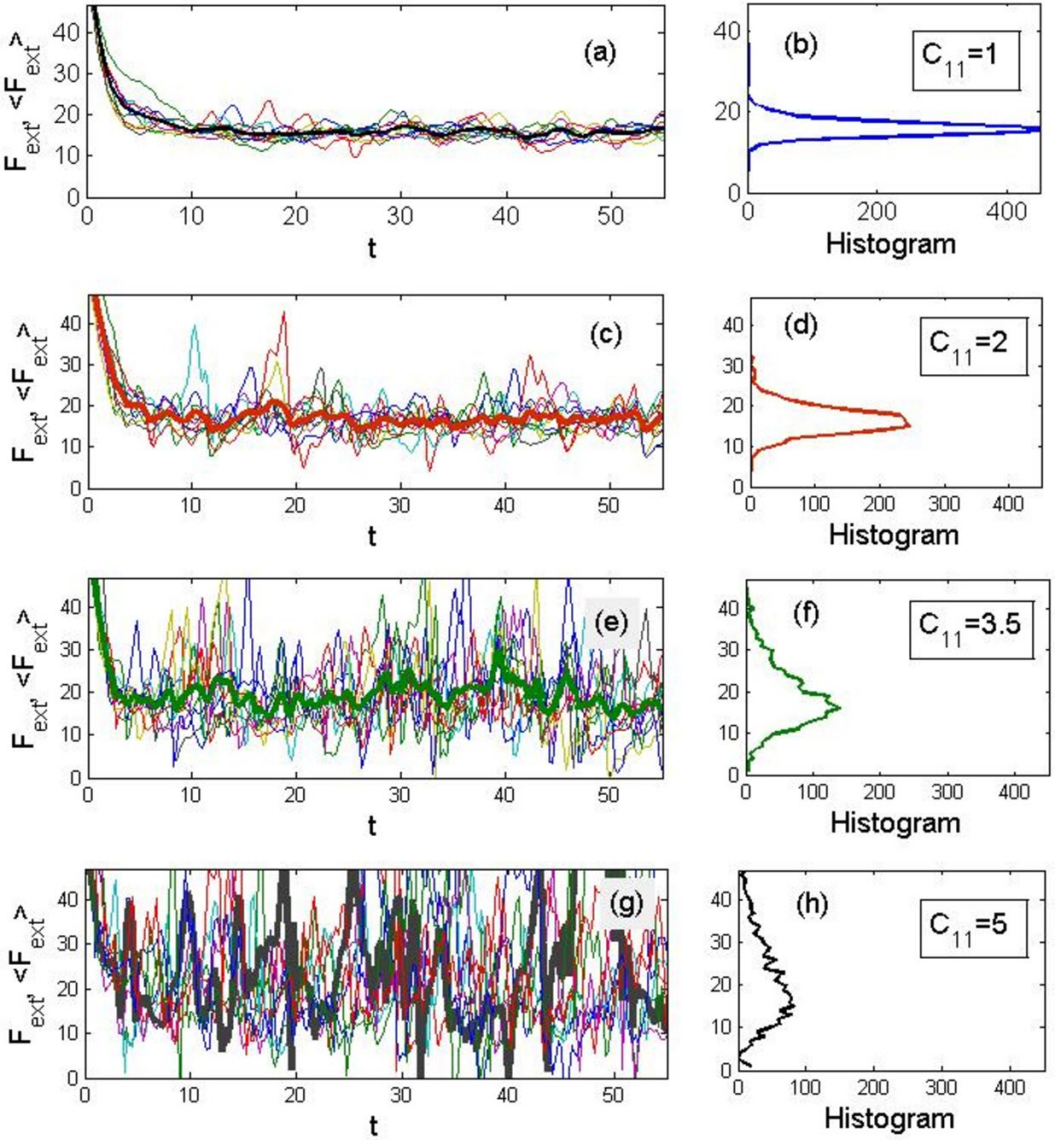
Results of averaging over ten implementations in four systems in the order of stiffness increase. On left-column panels (**a**), (**c**), (**e**) and (**g**), thin curves represent the families of force–time dependences at a constant velocity V = const, and the bold curve shows the result of averaging. On the right-column panels (**b**), (**d**), (**f**), and (**h**), corresponding histograms of the probability of detecting a given force F_ext value accumulated over all implementations for the entire observation interval are shown.

Since we cannot replicate exactly the experiment in numerical simulations, the homogenisation can be realised through the procedure of averaging over many implementations in a mesoscopic system that we are essentially dealing with here. In practice, this should correspond to the summation of contributions from various areas of a real body. Figure 6 shows the results of such an averaging over ten implementations in four systems of different stiffnesses. On its left-column panels (a), (c), (e) and (g), thin curves represent the families of force–time dependences (or shear, which is the same at a constant velocity $V=const$) and the bold curve shows the result of averaging. On the right-column panels (b), (d), (f), and (h), are corresponding histograms of the probability of detecting a given force ${F}_{ext}$ value accumulated over all implementations for the entire observation interval are given.

It can be noticed that the position of a maximum in each of the histograms (the most probable value of force) is virtually independent on the rigidity of a system, while a “tail” forms in the distribution, and hence the average (and therefore expected in a macroscopic system) value shifts towards increase. This shift is caused by relatively rare local force maxima attributed to the “stick–slip” phenomenon. Hence, it is plausible to believe that specifically these sharp maxima in rigid systems are responsible for the increase in measured macroscopic force. The background common to all the averaged curves comes from the viscous contributions to the motion resistance, which according to the model is present in all systems, i.e. hard, soft, and even “liquid”.

## Discussion

Computer simulations in Section.“Analysis of PWIT in a simple shear within the heuristic model framework” reveal that shear in a system of ordered particle domains realises by the movement of lattice defects executing the PWIT. The latter causes the sub-division of large domains into smaller disoriented fragments. The increase of ordering ranges in the system (enlargement of the original domains) leads to more chaotic behaviour in the system during shearing.

Such a system evolution resembles the process of fragmentation in metals during large plastic deformation. Namely, weakly-disoriented cells of tens-nanometre scale in size form at the initial stages of plastic deformation, developing a fine network of low-angle boundaries (LABs)^39–41^. With the increase of accumulated strain level, the boundaries gradually increase disorientations evolving into high-angle boundaries (HABs).

Such metal fragmentation processes are often described within the framework of a partial disclination model^42–44^. The disclinations can be interpreted as the sources of high elastic stresses that cause distortions and rotations of crystal lattice. Unlike the disclination model, PWIT reflects only the geometric side of the phenomena, and to calculate the fragmentation process, it is necessary to use variational principles. Such a situation is well illustrated in^22^, where PWIT is used in the study of shell stability. However, in some cases only the kinematic image of a phenomenon is also of interest and helps to put forward reasonable hypotheses about its nature. The presented here approach gives more freedom to study the related processes in dynamics. It helps to understand them better and to create new ideas, because it is based on relatively fast simulations and allows simple visualisation of the events in the system under consideration using standard MatLab codes. Such cases include the two SPD effects below that are much easier to investigate in a PWIT than in a partial disclination model.

In this work, we do not analyse directly thermal effects including adiabatic heating that may lead to the formation of e.g. adiabatic shear bands^45^. We assume rapid dissipation of thermal energy, and the ‘thermostat’ implemented in the model imposes a homogenous thermal field across the entire simulation volume. However, the temperature effects can be rather easily regulated by the intensity of noise applied in the equations of motion: from the practically frozen systems up to the melted ones. Thermal field along with associated atomic vibrations certainly affects all the patterns observed in numerical experiments. Among others, it influences stronger or weaker localisation of plastic deformations. We plan to collect and to publish the results related to the thermal effects accompanying plastic transformations in follow-up studies.

It is known that sliding along deformation induced HABs is possible even at cryogenic temperatures^46–49^. In such a case, deformation induced HABs can be PWIT elements. Sliding along them realises by the motion of grain boundary dislocations. The described vision of grain subdivision process can also be interpreted in terms of percolation theory (connections problem)^50^ as the sequential transformation of a lattice into increasing number of PWIT elements.

Let’s introduce a quantity $\Theta$ as a fraction of grid links belonging to PWIT. According to the percolation theory, such units are isolated random inclusions in the lattice under consideration while $\Theta <<1$. With the increase of $\Theta$, the links begin aggregating, i.e. form coherent clusters. When $\Theta$ reaches a critical value ${\Theta }_{c}$, a qualitative change occurs in the system. A so-called percolation cluster (PC) forms, which permeates the lattice across. The value ${\Theta }_{c}$ is then called a ‘percolation threshold’ for bond problem and depends on a lattice type, e.g., ${\Theta }_{c}=0.{65}$ for hexagonal lattice^50^.

Since PC permeates the lattice across, it ensures the transfer of isometric transformations through the entire representative volume of a material. From this point on, transformations $\Delta {{\varvec{P}}}_{i}$ in the mapping (4) can be realised primarily by sliding along the boundaries of PC. The latter has a fractal structure, with loops of varying scales^50^. The average size of loop sections $L$ is equal to the radius of correlation and is much larger than the size of the lattice cells $l$. For this reason, the loops appear smooth within $L$-scale. The magnitude of $L$ near the percolation threshold can be determined by formula^50^:

10 $$L=l{\left|\Theta -{\Theta }_{c}\right|}^{-\nu }$$

where $\nu$ is a correlation radius index. For two-dimensional problems, $\nu =1.33$.

**Figure 7:**
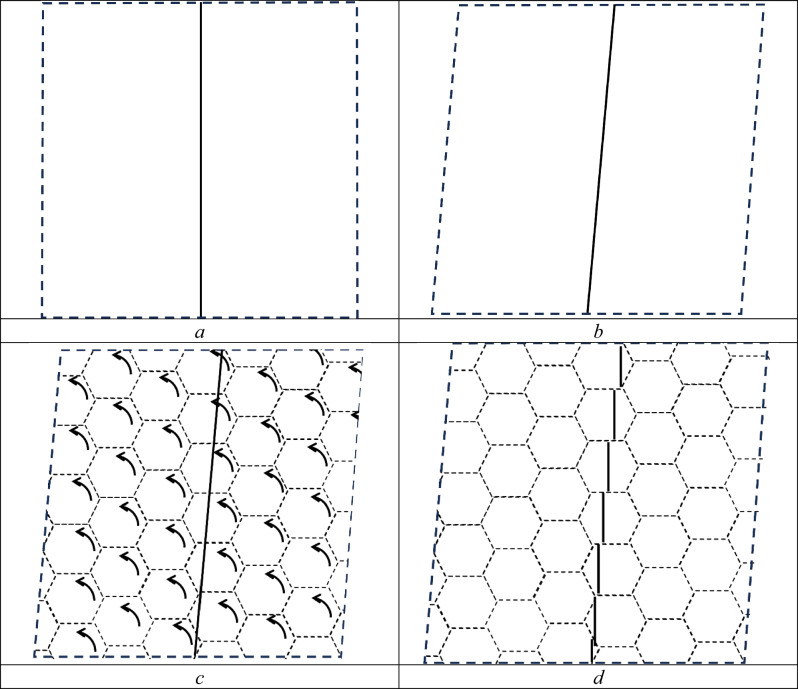
Diagram illustrating the realisation of a simple shear according to mapping (4) in the presence of a percolation cluster: (**a**) initial state; (**b**) the result of a transformation action ΔE (elastic deformation); (**c**) percolation cluster shown by dotted lines; d the result of transformation action ΔP based on shears along the boundaries of the percolation cluster.

Figure 7 schematically shows one step of a simple shear according to mapping (4) in the presence of a PC. The latter is presented as a hexagonal network with cell sizes $L$. It is easy to realise that the areas bounded by PC cells will rotate counter-clockwise to relax the elastic stresses produced by shear, Fig. 7. As can be seen from this figure, the isometry is preserved in these regions, and strain gradient in simple shear is achieved primarily by shears along PCs.

Note, the described model of shear percolation over hexagonal network in elastic medium superficially resembles the heuristic model of molecular vortices^51^, which James Clerk Maxwell used to explain his theory of the electromagnetic field.

In the above, we have considered only one step of a simple shear. All subsequent steps are similar, which ensures the stationarity (in the statistical sense) of the proposed transformation. It should also be noted that each step is related to the action of its own PC. The motion of grain boundary dislocations leads to a change in boundary structure, which may lead to a temporary termination in following shear. Apparently, this effect is reflected in our model as the ‘stick–slip’ behaviour of particles under shear, see previous section. Therefore, a formed PC may break in several places in one mapping step (4), and next step will require the formation a new PC. This means, a PWIT must have certain ‘stockpile’ of grid links to realise the proposed mechanism. In other words, the percolation by shear initiated at.

$\Theta ={\Theta }_{c}$ might stagnate at $\Theta ={\Theta }_{c1}>{\Theta }_{c}$.

It has been shown that steady-state deformation can be established at large strains by simple shear in HPT process^52^. Such a state is characterised by a statistically invariable sub-microcrystalline structure in a material and the absence (saturation) of strain hardening. It is interesting to note that in such a case, the fraction of HABs exceeds the value of ${\Theta }_{c}=0.65$, and the steady state emerges near the specified threshold. From our perspective, this enables putting forward a hypothesis that steady-state deformation should be attributed to the statistical stationarity of PWIT realising through the percolation of shear along high-angle boundaries.

Within the PWIT model framework, another interesting effect observed in HPT and known as SPD-induced accelerated mass transfer (diffusion) can also be explained. It refers to a dramatic increase (by many orders of magnitude) of the effective diffusion coefficient in metallic materials that undergo deformation by simple shear^53^. In the case of HPT processing of metallic materials, this effect is manifested by the rapid transfer of phase fragments in a deformable sample, e.g.^54^, leading to their mechanical intermixing^55^. In the works^33,56^, this effect is associated with the occurrence of a stochastic vortex motion in a deformable sample. Let us show how the above-mentioned random rotations of the PC cells can act in this capacity, see Fig. 7.

According to the presented model, these are formed by combining highly disoriented fragments (domains). The characteristic size of cells $L$ is much larger than the characteristic size of fragments $l$: $L=Cl, C>>1$. The cells rotate by sliding along their boundaries. Since specific HABs become “frozen” for some time immediately after the act of sliding (stick–slip effect), this mechanism requires the formation of an increasing number of rotations of highly disoriented fragments. Thus, during large deformation by shear, the fragments remain virtually unchanged while sequentially enter various random ‘rotation ensembles’. Let us estimate the angular velocity of their rotations $\omega$.

**Figure 8:**
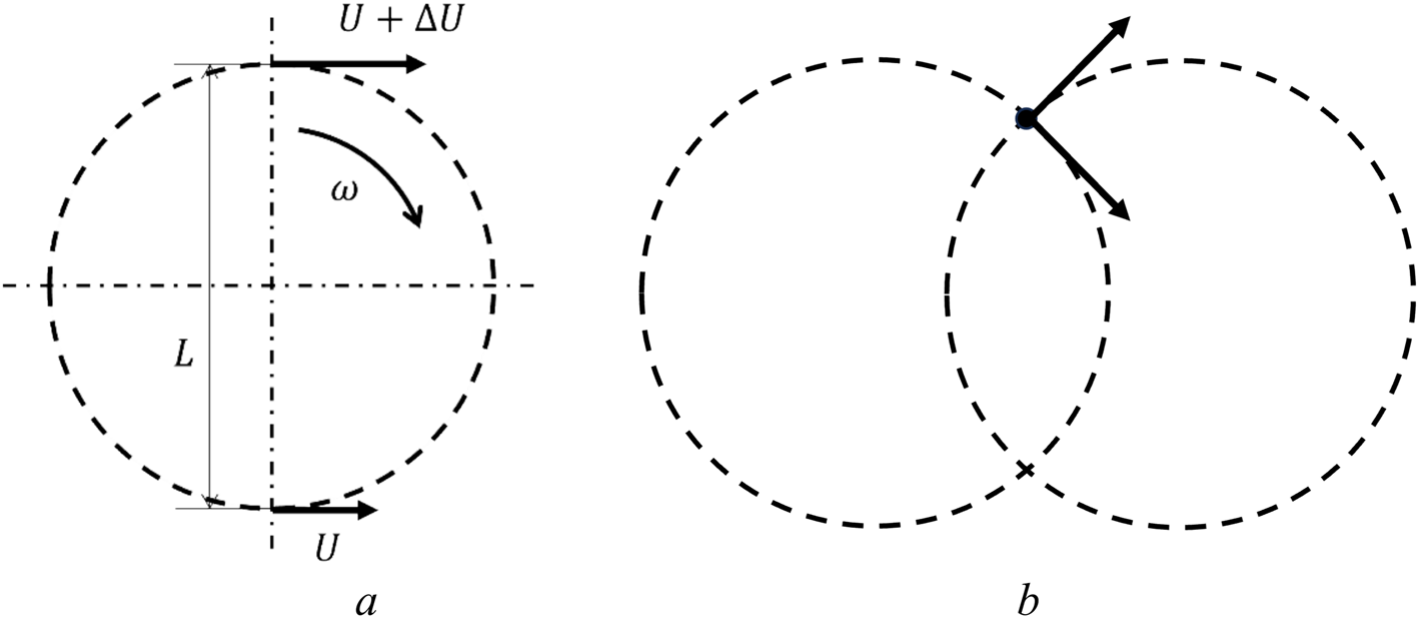
Schematics towards the estimate of magnitudes of angular (**a**) and pulsation (**b**) velocity.

From Fig. 8a, it follows that $\omega L=\Delta U$, i.e. $\omega =\dot{\gamma }$, where $\dot{\gamma }$ is shear strain rate, $\Delta U$ is change in the speed of movement $U$ at a scale $L$.

Let us consider an arbitrary fragment. Over time, the fragment may engage in various rotation ensembles passing through, Fig. 8b. Obviously, the value of pulsation velocity modulus is

11 $$v\approx \omega \frac{L}{2}=\frac{\dot{\gamma }\cdot L}{2}$$

**Figure 9:**
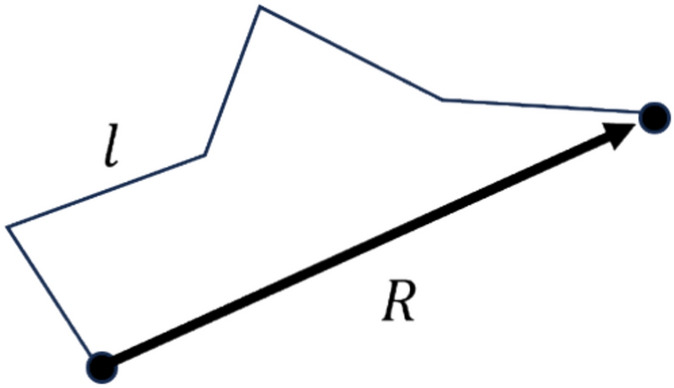
Fragment trajectory.

The direction of the pulsation velocity is assumed to be random. Then, the trajectory of fragment motion is a random broken line, as in self-diffusion, Fig. 9. It is also assumed that the direction of pulsation velocity is preserved while the point passes path $l$ at the rotation ensemble interface. This defines the length of a polyline segment.

The distance of a radius-vector points from the origin at the N-th and (N-1)-th steps are connected by an obvious relation:

12 $${\overrightarrow{R}}_{N}={\overrightarrow{R}}_{N-1}+\overrightarrow{l}$$

Hence

13 $${R}_{N}^{2}={R}_{N-1}^{2}+2\overrightarrow{l}{\overrightarrow{R}}_{N-1}+{l}^{2}$$

Upon averaging and accounting that each following step is not correlated with preceding ones, we obtain

14 $$\langle {R}_{N}^{2}\rangle =\langle {R}_{N-1}^{2}\rangle +{l}^{2}$$

where angular brackets indicate averaging over possible realisations. It follows that

15 $$\langle {R}_{N}^{2}\rangle =N{l}^{2}$$

Each step requires time

16 $$\Delta t=\frac{l}{v}=\frac{2l}{\dot{\gamma }\cdot L}$$

Therefore

17 $$N=\frac{t}{\Delta t}=\frac{L}{2l}\dot{\gamma }\cdot t$$

Substituting *N* in (15), we obtain:

18 $$\sqrt{\langle {R}_{N}^{2}\rangle }=\sqrt{\frac{Ll}{2}\dot{\gamma }\cdot t}$$

Given that $\dot{\gamma }\cdot t=\gamma$, from the last equation we obtain

19 $$\sqrt{\langle {R}_{N}^{2}\rangle }=\sqrt{\frac{Ll}{2}\gamma }=l\cdot \sqrt{\frac{C}{2}\gamma }$$

Assuming $l\approx 300 nm$ and $\gamma \approx \text{ 10}$ in aluminium alloys) and $C\approx 100$, we obtain that fragments can travel distances at the order of 10 μm, approximately order of magnitude more than their size.

Comparing (18) to a similar equation for diffusion in the second Fick’s law $\sqrt{\langle {R}_{N}^{2}\rangle }=\sqrt{2Dt}$, suggests the introduction of an ’effective diffusion coefficient’ ${D}_{\text{eff}}$ accounting for random rotations of fragments in simple-shear deformation (similar to the effective diffusion coefficient in turbulent flow)

20 $${D}_{\text{eff}}=\frac{Ll}{4}\dot{\gamma }=\frac{C{l}^{2}}{4}\dot{\gamma }$$

At $l\approx 1{0}^{-5}cm$ and $C\approx 100$, we obtain ${D}_{\text{eff}}\approx 2.5\cdot 1{0}^{-9}\dot{\gamma }\cdot c{m}^{2}{c}^{-1}$. For $\dot{\gamma }\approx\text{ 1}{c}^{-1}$ (characteristic value of shear strain rate in SPD), we obtain that the effective diffusion coefficient ${D}_{\text{eff}}$ can exceed the coefficient of thermally-only activated diffusion in a solid ${D}_{\text{l}}$ by seven (7!) orders of magnitude or even more. This may explain the abnormally rapid rate in the SPD-induced accelerated mass transfer.

## Conclusions

Piecewise isometric transformation (PWIT) has been introduced as a novel geometric approach for describing plastic deformations in crystalline materials. This approach has been shown to be more general than the theories widely used at present by enabling simpler universal solutions to multi-scale problems. The capacities of PWIT approach are illustrated by solving two important problems.

Steady-state stage of simple shear at large plastic deformation is explained by PWIT as rotations of sub-grain fragments along high-angle boundaries. In agreement with earlier works, our approach can explain the stabilisation of sub-grain sizes and the absence of strain hardening. Unlike earlier qualitative explanations of this phenomenon, though, the PWIT-based approach enables quantitative estimate of energy consumption by grain-boundary sliding at steady-state deformation.

SPD-induced accelerated diffusion (mass transfer) has been explained using the PWIT approach. The proposed mechanism is associated with sub-grain boundary sliding. Quantitative estimate of this process shows that effective diffusion coefficient ${D}_{\text{eff}}$ in severe simple-shear deformation can exceed thermally-activated diffusion coefficient by seven orders of magnitude or even more.

## Data Availability

All the data generated in the simulations can be made available upon request to A.F.
